# *Akkermansia muciniphila* May Determine Chondroitin Sulfate Ameliorating or Aggravating Osteoarthritis

**DOI:** 10.3389/fmicb.2017.01955

**Published:** 2017-10-09

**Authors:** Qi Wang, Shui-Qing Huang, Chang-Qing Li, Qin Xu, Qing-Ping Zeng

**Affiliations:** ^1^Clinical Pharmacology Institute, Guangzhou University of Chinese Medicine, Guangzhou, China; ^2^Basic Medical Science College, Guangzhou University of Chinese Medicine, Guangzhou, China; ^3^Tropical Medicine Institute, Guangzhou University of Chinese Medicine, Guangzhou, China

**Keywords:** chondroitin sulfate, osteoarthritis, gut microbiota, probiotic, antibiotic, opportunistic infection, pro-inflammation, anti-inflammation

## Abstract

Chondroitin sulfate (CS) has shown either ameliorating or aggravating effects on osteoarthritis (OA) in separately conducted clinical trials. Because CS is usually administered orally, it should be affected by or would impact on the individual gut microbiota. Evidence is accumulating that CS can nourish sulfatase-secreting bacteria (SSB) and sulfate-reducing bacteria (SRB). To decipher how can an individual gut microbiota determine the clinical values of CS for treatment on OA, we suggest here that CS would give distinct outcomes for OA treatment depending on *Akkermansia muciniphila*, a gut commensal probiotic bacterial species as optimal presence albeit also behaving as mucus-eroding bacteria (MEB) when abundant presence. Briefly, CS would ameliorate OA if *A. muciniphila* is present due to without overgrowth of SSB and SRB, whereas CS would aggravate OA if *A. muciniphila* is absent because of failure in or lack of competition with abundant SSB and SRB. By noting such a frequently ignored phenomenon, we urge the development of non-orally administering CS to minimize its side-effects and extend it to other medicinal applications.

Chondroitin sulfate (CS), also known as polysaccharide constituted of alternated residues of N-acetylgalactosamine and glucuronic acid, is a naturally O-sulfated glycan in cartilage and bones. CS extracted from cow or pig cartilage is prescribed as a symptomatic slow-acting drug for osteoarthritis (OA) in 22 countries, including the European Union, but it is still regulated in the USA as a dietary supplement for improving OA (Jordan, 2003). The United States Food and Drug Administration (FDA) denied a request that CS be labeled as reducing the risk of OA and OA-related joint pain, tenderness, and swelling (FDA, 2004). Despite compelling evidence that CS interferes with OA progression (Vergés and Castañeda-Hernández, 2004; Clegg et al., 2006; Reginster et al., 2007), a systematic review of 20 clinical trials concluded that CS had not demonstrated any symptomatic benefit and discouraged its use in routine clinical practice (Reichenbach et al., 2007). A recent Cochrane clinical trial review concluded that CS appeared to alleviate pain in the short term, but did not improve or maintain the health of joints affected by OA (Singh et al., 2015).

Because of this shortcoming in pain relief, CS was not recommended for the symptomatic treatment of knee OA (Jevseva et al., 2013). Combined treatment with CS and glucosamine sulfate or glucosamine hydrochloride did not reduce joint damage in a rabbit model of knee OA (Roman-Blas et al., 2017b). CS plus glucosamine sulfate was not superior to placebo in alleviating articular lesions in a randomized, double-blind, placebo-controlled clinical trial in patients with knee OA (Roman-Blas et al., 2017a).

The effectiveness, benefits, and harms of CS for treating OA are not fully understood, which is possibly attributed to a “good” or “bad” study design. Contamination of heparin with CS has been associated with adverse clinical events, such as allergy and hypertension (Kishimoto et al., 2008), and the potential effect of CS-mediated gut dysbiosis on the differential pharmaceutical outcomes of CS must be considered. Here we aim to give a perspective on the clinical assessment of the impact of CS on OA and other inflammatory disorders.

## Explaining inconsistent outcomes of CS on OA by the individual-specificity of gut microbiota symbionts

Considering the oral administration, gastrointestinal uptake, and structural similarity of CS with mucin-type *O*-glycans of the colonic epithelium, we propose a hypothesis of an *Akkermansia muciniphila*-dependent CS effect on OA, in which CS improves OA if *A. muciniphila* is present, and CS aggravates OA if *A. muciniphila* is absent. *A. muciniphila* is a species of mucin-eroding bacteria (MEB) that competes with sulfatase-secreting bacteria (SSB) and the population should increase when SSB are rare and decrease when SSB are abundant. MEB depend on mucin-type *O*-glycans as a sole carbon source; SSB are mucin generalists that can utilize other polysaccharides including those in dietary fiber (Desai et al., 2016).

Colonization of *A. muciniphila* in the gastrointestinal tract varies among individuals (Bressa et al., 2017), and affected by many variables including consumption of fruits and vegetables, drugs, obesity, and surgical history. *A. muciniphila* has been shown to increase following restriction in dietary fiber and a shift from fiber to mucin consumption (Desai et al., 2016) and increase of *Akkermansia* spp. has been associated with the antiobesity activity of a polyphenol-rich cranberry extract (Anhê et al., 2015). The antiobesity activity of capsaicin observed in mice fed a high-fat diet was also associated with increase of *A. muciniphila* (Shen et al., 2017). Enrichment of gut *A. muciniphila* populations has been observed in diabetes patients treated with metformin (de la Cuesta-Zuluaga et al., 2017), and following weight loss in obese and/or type 2 diabetes patients (Remely et al., 2016). Roux-en-Y gastric bypass surgery has also been found to increase *A. muciniphila* populations (Yan et al., 2016).

How can *A. muciniphila* residing in the gut confer a beneficial effect of CS on OA? Why is CS ineffective or even harmful when SSB preferentially occupy the gut? *A. muciniphila* may act as a gatekeeper of the mucosa (de Vos, 2017) or stimulate the mucosal immunity to prevent pathogenic invasion, and prompt thickening of the colonic mucosa to reduce the susceptibility to infection. Overgrowth of *A. muciniphila* is thought to cause mucin degradation, severe damage of the colonic mucosa, extensive endotoxin leakage, and unrecoverable tissue damage. *A. muciniphila* and *B. caccae* have been shown to stimulate epithelial access and initiate lethal colitis by the mucosal pathogen *Citrobacter rodentium* in gnotobiotic mice with a synthetic gut microbiota community (Desai et al., 2016).

Although a small *A*. *muciniphila* population may induce mucin turnover and a large population may induce colonic mucosal injury, a threshold level has not been identified. If bacterial consumption of mucin-derived nutrients exceeds production, then the integrity of the colonic barrier might be compromised (Rey et al., 2013). The overgrowth of *A. muciniphila* might derive from genetic immune deficiency. That is supported by the observation that *A. muciniphila* promoted intestinal inflammation in genetically susceptible germ-free Il10^−/−^ mice (Seregin et al., 2017).

## Evidence for the probiotic value of *A. muciniphila*

Evidence of a dose-dependent benefit of *A. muciniphila* on human health is lacking, but antibiotics capable of eliminating *Akkermansia* reinforce the colonic mucus barrier and prevent heme-dependent cytotoxic micelles from reaching the epithelium (Ijssennager et al., 2015). *A. muciniphila* may also be a novel functional microbe with probiotic properties (Dao et al., 2016). Evidence for the therapeutic values of *A. muciniphila* in metabolic diseases includes an inverse correlation with metabolic changes occurring with obesity and type 1 diabetes in mice and humans (Shin et al., 2014; Gomes et al., 2017) and prevention of obesity induced by changes in adipose tissue metabolism and gut barrier function caused by a high-fat diet in mice (Everard et al., 2013).

Favorable effects of *A. muciniphila* on improved glucose tolerance and maintained high interferon (IFN) γ levels for controlling various pathogen infections, primarily those caused by intracellular pathogens, have been described by Greer et al. (2016) and Ottman et al. (2017) attributed a positive correlation of *A*. *muciniphila* and gut health to immune modulatory effects and immune tolerance to this commensal bacterium. *A. muciniphila* has been reported to promote intestinal barrier integrity and ameliorate experimental alcoholic liver disease by enhancing colonic mucus thickness and tight-junction expression (Grander et al., 2017). *Bifidobacterium* that resemble *A. muciniphila* and degrade mucins by secreting endo-α-N-acetylgalactosaminidase and 1,2-α-L-fucosidase (Ruas-Madiedo et al., 2008) have probiotic behavior that might stimulate mucin overproduction and accelerate replenishment of lost mucins.

The anti-inflammatory activity of CS has been demonstrated *in vitro*. When CS was immobilized on a scaffold and exposed to proinflammatory cytokines or cytokine-stimulated peripheral blood mononuclear cells, significant increases in the production of molecules with immunosuppressive activity and the expression of their inducible enzymes were observed (Corradetti et al., 2016). Subcutaneous implantation of CS-immobilized scaffold in rats reduced leukocyte infiltration and upregulated genes encoding proteins associated with apoptosis of inflammatory cells (Corradetti et al., 2016). CS was found to suppress nuclear NF-κB translocation *in vitro* (Tan and Tabata, 2014), and to inhibit NF-κB activation by pathogen- and damage-associated molecules in cultured THP-1 macrophages (Stabler et al., 2017).

## Proinflammatory activity of CS-promoted SSB and anti- inflammatory activity of antibiotic-resistant *A. muciniphila*

CS supports the overgrowth of *Bacteroides* sp. strain 3452A (an unnamed DNA homology group), *B. ovatus*, and *B. thetaiotaomicron* (Lipeski et al., 1986) in the colon and can promote the growth of *B. thetaiotaomicron*, which is an SSB, and *Desulfovibrio piger*, a species of sulfate-reducing bacteria (SRB) (Rey et al., 2013). SRB may thus thrive if supported by a food chain-like lifestyle, i.e., SRB growth depends on sulfate-containing CS. A number of SSB species, including *B. thetaiotaomicron* J1 and 82, *B. ovitus* E3, and *Clostridium hathewayi* R4, have been isolated from healthy subjects (Shang et al., 2016). Theoretically, oral CS administration should support the proportional expansion of SSB and SRB in the distal gut.

**Figure 1:**
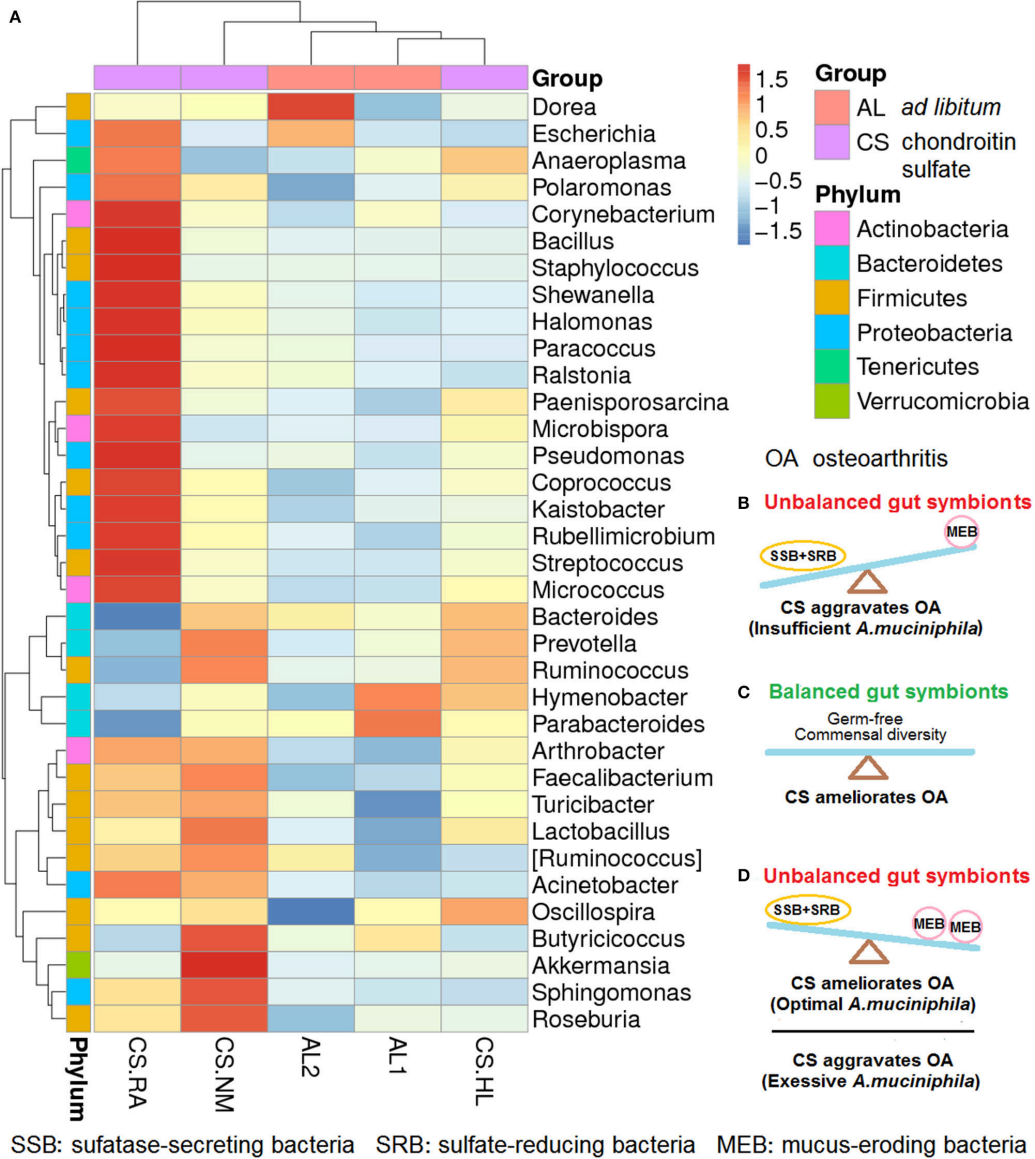
Changes in the gut microbiota profiles of mice fed *ad libitum* (AL) and chondroitin sulfate (CS) (*n* = 3 for mice fed CS; *n* = 2 for mice fed AL; CS.RA, CS.NM, and CS.HL represent three mice fed CS; AL1 and AL2 represent two mice fed AL). **(A)** CS-induced gut microbiota shifts that varied in individual mice. **(B)** Unbalanced gut symbionts with more SSB+SRB than MEB species that aggravate OA due to insufficient *A. muciniphila*; **(C)** balanced gut symbionts in germ-free conditions or with commensal diversity that ameliorate OA because of no gut microbiota interference. **(D)** Unbalanced gut symbionts with more MEB than SSB+SRB that ameliorate OA when the abundance of *A. muciniphila* is optimal or aggravate OA if the colonization of *A. muciniphila* is excessive. The relative abundance of bacteria is represented by red (increased) to blue (decreased) colors accounting for Z values from −1.5 to 1.5.

We previously reported that daily CS feeding alters the gut microbiota spectrum of mice, but the response varied with individual differences (Figure 1A). In mice with symptoms of early-phase rheumatoid arthritis (RA), *Bacillus cereus*, an SSB was abundant and *A. muciniphila* was rare compared with normal mice. CS.NM with a normal phenotype has more *A. muciniphila*, but less *B. cereus*. CS.HL suffering from slight hairless shows no overgrowth of *B. cereus* and *A. muciniphila* (Liao et al., 2017b). Distinct gut microflora communities might be rapidly established in mice fed CS at identical doses and schedules.

As shown in Figure 1B, when the proliferation of SSB plus SRB exceeds that of MEB because of unbalanced gut symbionts, CS would aggravate OA because of an *A. muciniphila* population insufficient to stimulate mucin induction and mucus repair. If the gut is kept permanently germ-free, or a commensal diversity of SSB, SRB, MEB, and other symbions is maintained, CS would ameliorate OA (Figure 1C). CS might also ameliorate OA in the presence of normal mucosal immunity and an optimum gut milieu including *A. muciniphila*. However, CS would aggravate OA given an excess of *A. muciniphila* in a gut with decreased mucosal immunity such as in the immunocompromised, elderly and ill, or patients who are immunosuppressed following organ transplantation (Figure 1D).

We previously reported that CS increased the abundance of *Rikenella*, a genus of SSB, and *Desulfovibrio*, a genus of SRB, but did not significantly change the abundance of *A. muciniphila*. Surprisingly, *Rikenella* and *Desulfovibrio* were both eradicated by cephalosporin, but *A. muciniphila* increased. Conversely, *Rikenella* and *Desulfovibrio* thrived on exposure to berberine, but *A. muciniphila* was eliminated by this antibiotic analog. Consequently, cephalosporin significantly mitigated colonic mucus lesions and delayed the early pathogenesis of dementia, steatohepatitis, and atherosclerosis. Berberine significantly aggravated colonic mucus lesions and enhanced multi-systemic pathogenesis (Liao et al., 2017a). It has also been observed that germ-free rats had thinner colonic mucus layers, suggesting that microbial colonization is required for mucin production (Desai et al., 2016).

CS has found to upregulate expression of secretory leukocyte protease inhibitor (SLPI), a marker of tissue damage, and mucin 1 (MUC1), a marker of mucus repair; cephalosporin decreased SLPI and MUC1 expression to normal levels (Liao et al., 2017a). The SRB that flourish in association with CS administration could cause a related increase in mucin degradation compared with mucin biosynthesis. In line with the study results, ablation of MUC2 secretion brings bacteria into close contact with the gut epithelium, leading to inflammation and colon cancer (Van der Sluis et al., 2006). The results support the assumption that SSB compete with MEB, and that CS significantly increases the abundance of SSB and restores the *A. muciniphila* population after SSB are killed by antibiotics.

A pro- and anti-inflammatory activities of CS might reflect the temporal and spatial changes of lipopolysaccharide (LPS) content. We previously reported that peripheral (serum and muscle) LPSs declined to normal levels, and that visceral (hepatic and cardiac tissues) LPSs were elevated to maintain a pro-inflammatory state, or adipose and cerebral LPSs declined to maintain an anti-inflammatory state (Liao et al., 2017a,b). Administration of of a relative high 1.2 mg/kg LPS dose led to anti-inflammatory peritoneal changes, and a relative low 0.25 mg/kg LPS dose caused pro-inflammatory visceral changes, suggesting that anti-LPS treatment reduced LPS in peritoneal but not visceral tissues (Gao et al., 2015). A trend of decrease of anti-inflammatory indicators in the serum following oral CS administration might thus lead to erroneous conclusions of the anti-inflammatory effects of CS.

## Perspectives

The composition of the gut microbiome influences the pro- and anti-inflammatory activity of CS on aggravation or amelioration of OA via compromise or reinforcement of the colonic mucus barrier. Individual gut microbiota spectra should be carefully considered when evaluating the *in vivo* effects of CS on OA. The contribution of *A.muciniphila* in relation to dietary and other etiological variables must be considered to when evaluating the therapeutic values of CS in OA.

Caution should be exercised when administering CS orally to elderly OA patients, who may have decreased mucosal immunity. Similarly, OA patients with a history of organ transplantation and using immunosuppressive drugs might not maintain an *A. muciniphila* population optimal for avoiding persistent colonic mucus erosion and prolonged OA deterioration.

Development of next-generation CS formulations such as injectable lyophilized CS preparations not only minimizes CS-related side-effects, but also expands the indication of CS to a broad range of inflammatory disorders including inflammatory bowel disease. Alternatively, it should be an option that CS is used in combination with probiotics, prebiotics, or emerging dietary intervention (Segarra et al., 2016; Holleran et al., 2017).

## Author contributions

QZ and QW wrote the manuscript. QX, CL, and SH critically reviewed the manuscript. All authors have read and approved the final version of the manuscript.

### Conflict of interest statement

The authors declare that the research was conducted in the absence of any commercial or financial relationships that could be construed as a potential conflict of interest.
